# (*E*)-Methyl 2-[(1,3-dioxoisoindolin-2-yl)meth­yl]-3-phenyl­acrylate

**DOI:** 10.1107/S1600536812028140

**Published:** 2012-06-27

**Authors:** D. Lakshmanan, S. Murugavel, D. Kannan, M. Bakthadoss

**Affiliations:** a Department of Physics, C. Abdul Hakeem College of Engineering and Technology, Melvisharam, Vellore 632 509, India; b Department of Physics, Thanthai Periyar Government Institute of Technology, Vellore 632 002, India; c Department of Organic Chemistry, University of Madras, Maraimalai Campus, Chennai 600 025, India

## Abstract

In the title compound, C_19_H_15_NO_4_, the isoindole ring system is essentially planar [maximum deviation = 0.011 (1) Å] and is oriented at a dihedral angle of 75.7 (1)° with respect to the phenyl ring. The mol­ecular conformation is stabilized by an intra­molecular C—H⋯O hydrogen bond. The crystal packing is stabilized by C—H⋯O hydrogen bonds, which generate zigzag chains along the *a* axis. The crystal packing is further stabilized by a C—H⋯π inter­action.

## Related literature

For background to the applications of isoindolinones, see: Pendrak *et al.* (1994 ▶); De Clerck (1995 ▶); Stowers (1996 ▶); Heaney & Shuhaibar (1995 ▶). For related structures, see: Kannan *et al.* (2012 ▶); Liang & Li (2006 ▶). For graph-set analysis, see: Bernstein *et al.* (1995 ▶).
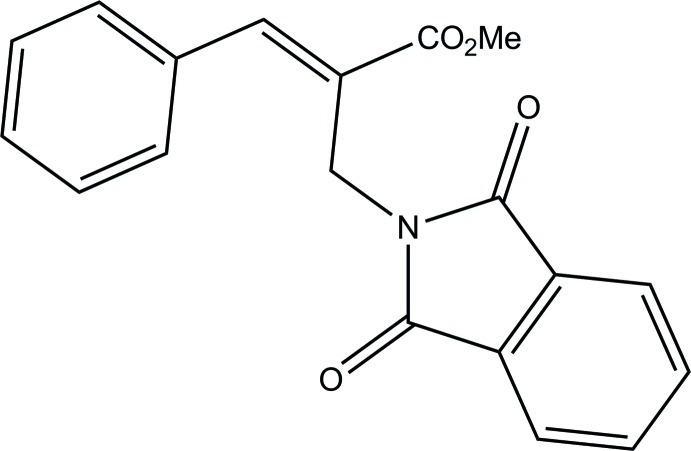


## Experimental

### 

#### Crystal data

C_19_H_15_NO_4_
*M* *_r_* = 321.32
Orthorhombic, 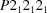

*a* = 8.682 (5) Å
*b* = 10.299 (4) Å
*c* = 17.903 (5) Å
*V* = 1600.8 (12) Å^3^
*Z* = 4
Mo *K*α radiation
μ = 0.09 mm^−1^
*T* = 293 K
0.24 × 0.22 × 0.17 mm

#### Data collection

Bruker APEXII CCD diffractometer
Absorption correction: multi-scan (*SADABS*; Sheldrick, 1996 ▶) *T*_min_ = 0.978, *T*_max_ = 0.984
18727 measured reflections
2694 independent reflections
2062 reflections with *I* > 2σ(*I*)
*R*_int_ = 0.032

#### Refinement

*R*[*F* ^2^ > 2σ(*F* ^2^)] = 0.039
*wR*(*F* ^2^) = 0.105
*S* = 1.02
2694 reflections
218 parameters
H-atom parameters constrained
Δρ_max_ = 0.14 e Å^−3^
Δρ_min_ = −0.17 e Å^−3^

### 

Data collection: *APEX2* (Bruker, 2004 ▶); cell refinement: *APEX2* and *SAINT* (Bruker, 2004 ▶); data reduction: *SAINT* and *XPREP* (Bruker, 2004 ▶); program(s) used to solve structure: *SHELXS97* (Sheldrick, 2008 ▶); program(s) used to refine structure: *SHELXL97* (Sheldrick, 2008 ▶); molecular graphics: *ORTEP-3* (Farrugia, 1997 ▶); software used to prepare material for publication: *SHELXL97* and *PLATON* (Spek, 2009 ▶).

## Supplementary Material

Crystal structure: contains datablock(s) global, I. DOI: 10.1107/S1600536812028140/go2060sup1.cif

Structure factors: contains datablock(s) I. DOI: 10.1107/S1600536812028140/go2060Isup2.hkl

Supplementary material file. DOI: 10.1107/S1600536812028140/go2060Isup3.cml

Additional supplementary materials:  crystallographic information; 3D view; checkCIF report

## Figures and Tables

**Table 1:** Hydrogen-bond geometry (Å, °) *Cg* is the centroid of the C2–C7 benzene ring.

*D*—H⋯*A*	*D*—H	H⋯*A*	*D*⋯*A*	*D*—H⋯*A*
C15—H15⋯O1	0.93	2.54	3.385 (3)	150
C9—H9*A*⋯O1^i^	0.97	2.59	3.217 (3)	122
C13—H13⋯*Cg* ^ii^	0.93	2.88	3.527 (3)	128

Symmetry codes: (i) 
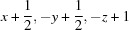
; (ii) 
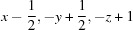
.

**Fig. 1.**
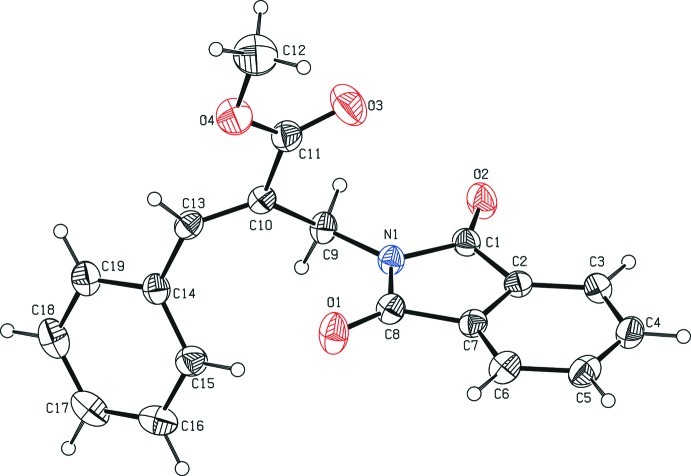
The molecular structure of the title compound with the atom numbering scheme. Displacement ellipsoids are drawn at the 30% probability level. H atoms are presented as a small circles of arbitrary radius.

**Fig. 2.**
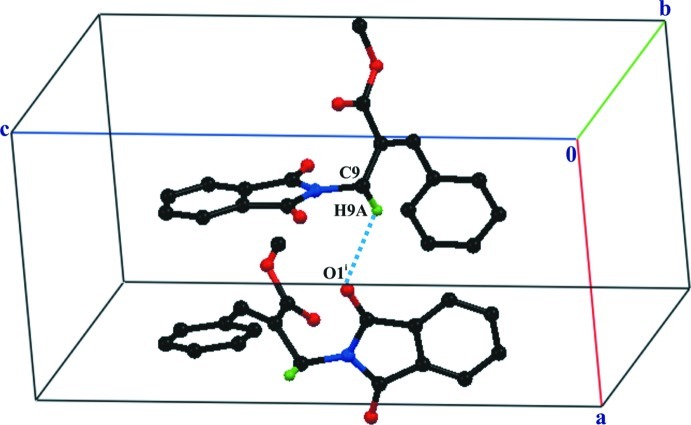
Part of the crystal structure of (I) showing intermolecular C—H···O hydrogen bonds (dotted lines), forming C(5) zigzag chains along the a axis. For clarity H atoms involved in the hydrogen bonds are shown. [Symmetry codes:(i)1/2 + x, 1/2 - y, 1 - z; (iii)1 + x, y, z; (iv)3/2 + x, 1/2 - y, 1 - z].

**Fig. 3.**
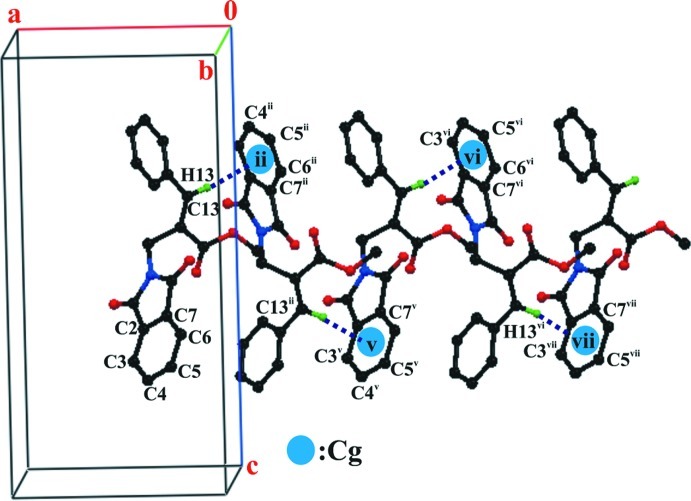
A view of the C—H···π interactions (dotted lines) in the crystal structure of the title compound, showing the formation of a chain along the a axis. Cg denotes centroid of the C2–C7 benzene ring. [Symmetry codes: (ii)-1/2 + x, 1/2 - y, 1 - z; (v)-1 + x, y, z; (vi)-3/2 + x, 1/2 - y, 1 - z; (vii)-2 + x, y, z].

## supplementary crystallographic information

### Comment

Isoindolinones and their derivatives have been investigated widely due to their profound physiological and chemotherapeutic properties. Many compounds containing the isoindolinone skeleton have shown antiviral, antileukemic, antiinflammatory, antipsychotic and antiulcer properties (Pendrak *et al.*, 1994; De Clerck, 1995). Isoindolinones are useful for the synthesis of various drugs and naturally occurring compounds (Stowers, 1996; Heaney & Shuhaibar, 1995).

Fig. 1. shows a displacement ellipsoid plot of (I), with the atom numbering scheme. The isoindole ring system is essentially planar [maximum deviation = 0.011 (2) Å for the C8 atom] and is oriented at a dihedral angle of 75.7 (1)° with respect to the benzene ring. The methyl acrylate (O3/O4/C10–C14) plane forms dihedral angles of 79.97 (7)° and 57.05 (9)° respectively, with the isoindole and benzene rings. The sum of bond angles around N1 (360.0°) indicates that N1 is in *sp*^2^ hybridization. The keto atoms O1 and O2 deviate by 0.023 (2) and -0.022 (2) Å, respectively, from the isoindole ring. The geometric parameters of the title molecule agree well with those reported for similar structures (Kannan *et al.*, 2012, Liang & Li 2006).

The molecular structure is stabilized by C15—H15···O1 intramolecular hydrogen bond, forming S(9) ring motif, (Bernstein *et al.*, 1995,) (Table 1). The crystal packing is stabilised by the intermolecular C9—H9A···O1(1/2+ x, 1/2-y,1-z), hydrogen bond which forms a C(5) zigzag chains along the *a* axis (Fig. 2). The crystal packing is further stabilised by C—H···π interactions, between H13 atom and the benzene ring (C2–C7) of an adjacent molecule, with a C13—H13···*Cg*(-1/2+X,1/2-Y,1-Z) separation of 2.88 Å, forming a chain along the *a* axis.(Table 1 and Fig. 3; *Cg* is the centroid of the (C2–C7) benzene ring. symmetry codes as in Fig. 3).

### Experimental

A solution of 2,3-dihydro-1*H*-isoindole-1,3-dione (1 mmol, 0.147 g) and potassium carbonate (1.5 mmol, 0.207 g) in acetonitrile as solvent was stirred for 15 minutes at room temperature. To this solution, methyl (2*Z*)-2-(bromomethyl)-3-phenylprop-2-enoate (1 mmol, 0.254 g) was added till the addition is complete. After the completion of the reaction as indicated by TLC, acetonitrile solvent was evaporated. Ethylacetate (15 ml) and water (15 ml) were added to the crude mass. The organic layer was dried over anhydrous sodium sulfate. Removal of solvent led to the crude product, which was purified through pad of silica gel (100–200 mesh) using ethylacetate and hexanes (1:9) as solvents. The pure title compound was obtained as a colorless solid (0.3105 g, 96% yield). Recrystallization was carried out using ethylacetate as solvent.

### Refinement

H atoms were positioned geometrically, with C—H = 0.93–0.98 Å and constrained to ride on their parent atom, with *U*_iso_(H)=1.5*U*_eq_ for methyl H atoms and 1.2*U*_eq_(C) for other H atoms. Friedel pairs were merged.

### Crystal data

**Table:**

C_19_H_15_NO_4_	*F*(000) = 672
*M**_r_* = 321.32	*D*_x_ = 1.333 Mg m^−^^3^
Orthorhombic, *P*2_1_2_1_2_1_	Mo *K*α radiation, λ = 0.71073 Å
Hall symbol: P 2ac 2ab	Cell parameters from 4756 reflections
*a* = 8.682 (5) Å	θ = 2.3–30.2°
*b* = 10.299 (4) Å	µ = 0.09 mm^−^^1^
*c* = 17.903 (5) Å	*T* = 293 K
*V* = 1600.8 (12) Å^3^	Block, colourless
*Z* = 4	0.24 × 0.22 × 0.17 mm

### Data collection

**Table:**

Bruker APEXII CCD diffractometer	2694 independent reflections
Radiation source: fine-focus sealed tube	2062 reflections with *I* > 2σ(*I*)
Graphite monochromator	*R*_int_ = 0.032
Detector resolution: 10.0 pixels mm^-1^	θ_max_ = 30.2°, θ_min_ = 2.3°
ω scans	*h* = −7→12
Absorption correction: multi-scan (*SADABS*; Sheldrick, 1996)	*k* = −14→14
*T*_min_ = 0.978, *T*_max_ = 0.984	*l* = −25→23
18727 measured reflections	

### Refinement

**Table:**

Refinement on *F*^2^	Primary atom site location: structure-invariant direct methods
Least-squares matrix: full	Secondary atom site location: difference Fourier map
*R*[*F*^2^ > 2σ(*F*^2^)] = 0.039	Hydrogen site location: inferred from neighbouring sites
*wR*(*F*^2^) = 0.105	H-atom parameters constrained
*S* = 1.02	*w* = 1/[σ^2^(*F*_o_^2^) + (0.0531*P*)^2^ + 0.1411*P*] where *P* = (*F*_o_^2^ + 2*F*_c_^2^)/3
2694 reflections	(Δ/σ)_max_ < 0.001
218 parameters	Δρ_max_ = 0.14 e Å^−^^3^
0 restraints	Δρ_min_ = −0.17 e Å^−^^3^

### Special details

**Table:**

Geometry. All esds (except the esd in the dihedral angle between two l.s. planes) are estimated using the full covariance matrix. The cell esds are taken into account individually in the estimation of esds in distances, angles and torsion angles; correlations between esds in cell parameters are only used when they are defined by crystal symmetry. An approximate (isotropic) treatment of cell esds is used for estimating esds involving l.s. planes.
Refinement. Refinement of F^2^ against ALL reflections. The weighted R-factor wR and goodness of fit S are based on F^2^, conventional R-factors R are based on F, with F set to zero for negative F^2^. The threshold expression of F^2^ > 2sigma(F^2^) is used only for calculating R-factors(gt) etc. and is not relevant to the choice of reflections for refinement. R-factors based on F^2^ are statistically about twice as large as those based on F, and R- factors based on ALL data will be even larger.

### Fractional atomic coordinates and isotropic or equivalent isotropic displacement parameters (Å^2^)

**Table:**

	*x*	*y*	*z*	*U*_iso_*/*U*_eq_	
C1	0.4553 (2)	0.43112 (18)	0.59855 (10)	0.0420 (4)	
C2	0.4172 (2)	0.34803 (18)	0.66357 (10)	0.0409 (4)	
C3	0.4672 (3)	0.3534 (2)	0.73676 (11)	0.0507 (5)	
H3	0.5364	0.4165	0.7527	0.061*	
C4	0.4100 (3)	0.2611 (2)	0.78517 (11)	0.0568 (5)	
H4	0.4416	0.2621	0.8348	0.068*	
C5	0.3071 (3)	0.1671 (2)	0.76190 (12)	0.0543 (5)	
H5	0.2702	0.1067	0.7961	0.065*	
C6	0.2582 (2)	0.1617 (2)	0.68843 (11)	0.0508 (5)	
H6	0.1895	0.0983	0.6723	0.061*	
C7	0.3151 (2)	0.25355 (18)	0.64031 (10)	0.0408 (4)	
C8	0.2867 (2)	0.27202 (19)	0.55914 (10)	0.0432 (4)	
C9	0.3800 (2)	0.4375 (2)	0.46398 (10)	0.0420 (4)	
H9A	0.4478	0.3857	0.4329	0.050*	
H9B	0.4244	0.5236	0.4680	0.050*	
C10	0.22571 (19)	0.44775 (17)	0.42645 (10)	0.0395 (4)	
C11	0.1140 (2)	0.5312 (2)	0.46622 (11)	0.0501 (5)	
C12	−0.1422 (3)	0.6087 (3)	0.47327 (18)	0.0915 (10)	
H12A	−0.1475	0.5854	0.5251	0.137*	
H12B	−0.2407	0.5942	0.4503	0.137*	
H12C	−0.1151	0.6988	0.4688	0.137*	
C13	0.1936 (2)	0.39586 (17)	0.35978 (10)	0.0417 (4)	
H13	0.0985	0.4174	0.3394	0.050*	
C14	0.2911 (2)	0.30857 (18)	0.31481 (10)	0.0424 (4)	
C15	0.3622 (2)	0.19997 (19)	0.34530 (12)	0.0507 (5)	
H15	0.3510	0.1820	0.3959	0.061*	
C16	0.4498 (3)	0.1182 (2)	0.30087 (14)	0.0616 (6)	
H16	0.4970	0.0458	0.3218	0.074*	
C17	0.4671 (3)	0.1438 (3)	0.22615 (14)	0.0654 (6)	
H17	0.5275	0.0895	0.1967	0.078*	
C18	0.3959 (3)	0.2488 (2)	0.19479 (13)	0.0626 (6)	
H18	0.4072	0.2652	0.1440	0.075*	
C19	0.3068 (3)	0.3311 (2)	0.23827 (11)	0.0530 (5)	
H19	0.2573	0.4015	0.2164	0.064*	
N1	0.37333 (17)	0.37951 (15)	0.53831 (8)	0.0399 (3)	
O1	0.20532 (18)	0.20880 (16)	0.51830 (8)	0.0641 (4)	
O2	0.53828 (19)	0.52473 (14)	0.59466 (8)	0.0606 (4)	
O3	0.1451 (2)	0.5935 (2)	0.52064 (11)	0.0886 (7)	
O4	−0.02687 (17)	0.53002 (17)	0.43641 (9)	0.0658 (5)	

### Atomic displacement parameters (Å^2^)

**Table:**

	*U*^11^	*U*^22^	*U*^33^	*U*^12^	*U*^13^	*U*^23^
C1	0.0396 (9)	0.0411 (9)	0.0454 (9)	0.0013 (8)	−0.0047 (8)	−0.0045 (8)
C2	0.0394 (8)	0.0399 (9)	0.0435 (9)	0.0080 (8)	−0.0025 (7)	−0.0032 (8)
C3	0.0580 (11)	0.0489 (11)	0.0452 (10)	0.0061 (10)	−0.0080 (9)	−0.0078 (9)
C4	0.0693 (13)	0.0614 (13)	0.0396 (9)	0.0168 (12)	−0.0047 (10)	0.0001 (9)
C5	0.0545 (11)	0.0588 (12)	0.0494 (10)	0.0087 (11)	0.0043 (9)	0.0114 (10)
C6	0.0439 (9)	0.0555 (11)	0.0530 (11)	−0.0011 (9)	−0.0005 (8)	0.0081 (10)
C7	0.0354 (8)	0.0444 (9)	0.0425 (9)	0.0058 (8)	−0.0006 (7)	0.0002 (8)
C8	0.0352 (8)	0.0475 (10)	0.0467 (9)	−0.0046 (8)	−0.0023 (8)	0.0022 (8)
C9	0.0369 (8)	0.0505 (10)	0.0387 (8)	−0.0063 (8)	−0.0016 (7)	0.0022 (8)
C10	0.0365 (8)	0.0399 (9)	0.0420 (9)	−0.0011 (7)	−0.0008 (7)	0.0035 (8)
C11	0.0479 (10)	0.0533 (11)	0.0492 (10)	0.0052 (9)	−0.0016 (9)	−0.0008 (10)
C12	0.0606 (14)	0.113 (2)	0.101 (2)	0.0375 (17)	0.0108 (15)	−0.0164 (19)
C13	0.0356 (8)	0.0440 (9)	0.0455 (9)	−0.0002 (8)	−0.0038 (8)	0.0053 (8)
C14	0.0372 (9)	0.0462 (10)	0.0436 (9)	−0.0073 (8)	−0.0039 (8)	−0.0027 (8)
C15	0.0542 (11)	0.0455 (10)	0.0522 (11)	−0.0016 (9)	0.0010 (9)	0.0021 (9)
C16	0.0587 (12)	0.0438 (11)	0.0823 (16)	0.0003 (10)	0.0047 (12)	−0.0083 (11)
C17	0.0635 (13)	0.0602 (14)	0.0726 (15)	−0.0085 (12)	0.0146 (12)	−0.0235 (12)
C18	0.0661 (14)	0.0739 (15)	0.0478 (11)	−0.0176 (13)	0.0066 (10)	−0.0123 (11)
C19	0.0552 (11)	0.0586 (12)	0.0451 (10)	−0.0080 (11)	−0.0029 (9)	−0.0016 (9)
N1	0.0370 (7)	0.0431 (8)	0.0397 (7)	−0.0047 (7)	−0.0047 (6)	−0.0003 (6)
O1	0.0650 (9)	0.0741 (10)	0.0532 (8)	−0.0324 (9)	−0.0156 (8)	0.0079 (7)
O2	0.0744 (10)	0.0536 (8)	0.0537 (8)	−0.0230 (8)	−0.0122 (8)	−0.0021 (7)
O3	0.0753 (11)	0.1053 (16)	0.0851 (12)	0.0247 (12)	−0.0146 (10)	−0.0455 (12)
O4	0.0431 (7)	0.0809 (11)	0.0734 (10)	0.0152 (8)	−0.0009 (7)	−0.0119 (9)

### Geometric parameters (Å, º)

**Table:**

C1—O2	1.206 (2)	C10—C11	1.479 (3)
C1—N1	1.397 (2)	C11—O3	1.198 (3)
C1—C2	1.482 (3)	C11—O4	1.334 (3)
C2—C7	1.381 (3)	C12—O4	1.448 (3)
C2—C3	1.382 (3)	C12—H12A	0.9600
C3—C4	1.379 (3)	C12—H12B	0.9600
C3—H3	0.9300	C12—H12C	0.9600
C4—C5	1.381 (3)	C13—C14	1.474 (3)
C4—H4	0.9300	C13—H13	0.9300
C5—C6	1.383 (3)	C14—C15	1.389 (3)
C5—H5	0.9300	C14—C19	1.396 (3)
C6—C7	1.372 (3)	C15—C16	1.385 (3)
C6—H6	0.9300	C15—H15	0.9300
C7—C8	1.486 (3)	C16—C17	1.372 (3)
C8—O1	1.208 (2)	C16—H16	0.9300
C8—N1	1.389 (2)	C17—C18	1.367 (3)
C9—N1	1.460 (2)	C17—H17	0.9300
C9—C10	1.502 (3)	C18—C19	1.386 (3)
C9—H9A	0.9700	C18—H18	0.9300
C9—H9B	0.9700	C19—H19	0.9300
C10—C13	1.337 (2)		
			
O2—C1—N1	124.33 (18)	O3—C11—C10	123.70 (19)
O2—C1—C2	129.82 (17)	O4—C11—C10	113.81 (18)
N1—C1—C2	105.84 (15)	O4—C12—H12A	109.5
C7—C2—C3	121.07 (19)	O4—C12—H12B	109.5
C7—C2—C1	108.26 (15)	H12A—C12—H12B	109.5
C3—C2—C1	130.67 (19)	O4—C12—H12C	109.5
C4—C3—C2	117.1 (2)	H12A—C12—H12C	109.5
C4—C3—H3	121.5	H12B—C12—H12C	109.5
C2—C3—H3	121.5	C10—C13—C14	127.70 (16)
C3—C4—C5	121.76 (19)	C10—C13—H13	116.2
C3—C4—H4	119.1	C14—C13—H13	116.2
C5—C4—H4	119.1	C15—C14—C19	118.41 (19)
C4—C5—C6	120.9 (2)	C15—C14—C13	122.11 (17)
C4—C5—H5	119.5	C19—C14—C13	119.39 (18)
C6—C5—H5	119.5	C16—C15—C14	120.5 (2)
C7—C6—C5	117.3 (2)	C16—C15—H15	119.8
C7—C6—H6	121.4	C14—C15—H15	119.8
C5—C6—H6	121.4	C17—C16—C15	120.2 (2)
C6—C7—C2	121.87 (17)	C17—C16—H16	119.9
C6—C7—C8	129.99 (18)	C15—C16—H16	119.9
C2—C7—C8	108.13 (16)	C18—C17—C16	120.2 (2)
O1—C8—N1	125.72 (17)	C18—C17—H17	119.9
O1—C8—C7	128.32 (18)	C16—C17—H17	119.9
N1—C8—C7	105.95 (15)	C17—C18—C19	120.4 (2)
N1—C9—C10	113.66 (15)	C17—C18—H18	119.8
N1—C9—H9A	108.8	C19—C18—H18	119.8
C10—C9—H9A	108.8	C18—C19—C14	120.3 (2)
N1—C9—H9B	108.8	C18—C19—H19	119.9
C10—C9—H9B	108.8	C14—C19—H19	119.9
H9A—C9—H9B	107.7	C8—N1—C1	111.81 (15)
C13—C10—C11	121.68 (17)	C8—N1—C9	126.31 (15)
C13—C10—C9	123.85 (17)	C1—N1—C9	121.87 (15)
C11—C10—C9	114.24 (16)	C11—O4—C12	116.5 (2)
O3—C11—O4	122.5 (2)		
			
O2—C1—C2—C7	179.2 (2)	C11—C10—C13—C14	−178.15 (18)
N1—C1—C2—C7	−0.7 (2)	C9—C10—C13—C14	7.6 (3)
O2—C1—C2—C3	−1.0 (3)	C10—C13—C14—C15	48.2 (3)
N1—C1—C2—C3	179.0 (2)	C10—C13—C14—C19	−135.2 (2)
C7—C2—C3—C4	−0.4 (3)	C19—C14—C15—C16	1.7 (3)
C1—C2—C3—C4	179.94 (19)	C13—C14—C15—C16	178.30 (18)
C2—C3—C4—C5	−0.1 (3)	C14—C15—C16—C17	0.0 (3)
C3—C4—C5—C6	0.5 (3)	C15—C16—C17—C18	−1.1 (3)
C4—C5—C6—C7	−0.5 (3)	C16—C17—C18—C19	0.6 (3)
C5—C6—C7—C2	0.0 (3)	C17—C18—C19—C14	1.1 (3)
C5—C6—C7—C8	179.04 (19)	C15—C14—C19—C18	−2.2 (3)
C3—C2—C7—C6	0.4 (3)	C13—C14—C19—C18	−178.92 (18)
C1—C2—C7—C6	−179.85 (17)	O1—C8—N1—C1	−179.99 (19)
C3—C2—C7—C8	−178.81 (18)	C7—C8—N1—C1	0.4 (2)
C1—C2—C7—C8	0.95 (19)	O1—C8—N1—C9	1.2 (3)
C6—C7—C8—O1	0.4 (3)	C7—C8—N1—C9	−178.40 (17)
C2—C7—C8—O1	179.5 (2)	O2—C1—N1—C8	−179.76 (18)
C6—C7—C8—N1	−179.94 (19)	C2—C1—N1—C8	0.2 (2)
C2—C7—C8—N1	−0.83 (19)	O2—C1—N1—C9	−0.9 (3)
N1—C9—C10—C13	−123.38 (19)	C2—C1—N1—C9	179.02 (16)
N1—C9—C10—C11	62.0 (2)	C10—C9—N1—C8	42.5 (3)
C13—C10—C11—O3	−169.5 (2)	C10—C9—N1—C1	−136.12 (18)
C9—C10—C11—O3	5.3 (3)	O3—C11—O4—C12	0.1 (4)
C13—C10—C11—O4	11.2 (3)	C10—C11—O4—C12	179.4 (2)
C9—C10—C11—O4	−174.02 (17)		

### Hydrogen-bond geometry (Å, º)

**Table:**

*D*—H···*A*	*D*—H	H···*A*	*D*···*A*	*D*—H···*A*
C15—H15···O1	0.93	2.54	3.385 (3)	150
C9—H9*A*···O1^i^	0.97	2.59	3.217 (3)	122
C13—H13···*Cg*^ii^	0.93	2.88	3.527 (3)	128

Symmetry codes: (i) *x*+1/2, −*y*+1/2, −*z*+1; (ii) *x*−1/2, −*y*+1/2, −*z*+1.
